# The development of a high-affinity conformation-sensitive antibody mimetic using a biocompatible copolymer carrier (iBody)

**DOI:** 10.1016/j.jbc.2021.101342

**Published:** 2021-10-25

**Authors:** Kristyna Blažková, Jana Beranová, Martin Hradilek, Libor Kostka, Vladimír Šubr, Tomáš Etrych, Pavel Šácha, Jan Konvalinka

**Affiliations:** 1 Institute of Organic Chemistry and Biochemistry, Czech Academy of Sciences, Prague, Czech Republic; 2 Department of Cell and Developmental Biology, Faculty of Science, Charles University, Prague, Czech Republic; 3 First Medical Faculty, Charles University, Prague, Czech Republic; 4 Institute of Macromolecular Chemistry, Czech Academy of Sciences, Prague, Czech Republic

**Keywords:** antibody mimetics, bicyclic phage display, molecular recognition, HPMA copolymer, PSMA, phage display, cyclic peptide, protein targeting, nanotechnology, chemical biology, HPMA, *N*-(2-hydroxypropyl)methacrylamide, 2-PMPA, 2-(phosphonomethyl)pentane-1,5-dioic acid, PSMA, prostate-specific membrane antigen, SPR, surface plasmon resonance, TATA, 1,3,5-triacryloyl-1,3,5-triazinane, TBMB, 1,3,5-tris(bromomethyl)benzene, TBS, Tris-buffered saline

## Abstract

Peptide display methods are a powerful tool for discovering new ligands of pharmacologically relevant targets. However, the selected ligands often suffer from low affinity. Using phage display, we identified a new bicyclic peptide binder of prostate-specific membrane antigen (PSMA), a metalloprotease frequently overexpressed in prostate cancer. We show that linking multiple copies of a selected low-affinity peptide to a biocompatible water-soluble *N*-(2-hydroxypropyl)methacrylamide copolymer carrier (iBody) improved binding of the conjugate by several orders of magnitude. Furthermore, using ELISA, enzyme kinetics, confocal microscopy, and other approaches, we demonstrate that the resulting iBody can distinguish between different conformations of the target protein. The possibility to develop stable, fully synthetic, conformation-selective antibody mimetics has potential applications for molecular recognition, diagnosis and treatment of many pathologies. This strategy could significantly contribute to more effective drug discovery and design.

The use of antibodies specific to pharmacologically relevant targets remains a gold standard in the development of therapeutic and diagnostic agents. Although antibodies tend to be more stable than most biologically active proteins, they are still sensitive to changes in the environment, such as fluctuations in temperature and pH. Furthermore, antibodies can be difficult to chemically modify and have a limited shelf-life. Potential batch-to-batch variability and limited tissue penetration also are among the reasons why researchers are exploring potential alternatives.

Multiple protein and nonprotein antibody mimetics have been designed. Alternative scaffolds such as knottins, aptamers, and affibodies, among others, are already well established (1).

Recently, we introduced iBodies (2), hydrophilic polymer-based antibody mimetics that comprise target-specific small-molecule ligands conjugated to a *N*-(2-hydroxypropyl)methacrylamide (HPMA)-based copolymer backbone. Initially designed to target active sites of enzymes using known inhibitors (3, 4, 5), iBodies have since been adapted for molecular recognition of targets without known enzymatic activity, such as His tag (6).

The most challenging task in developing antibody mimetics is to obtain a molecule capable of targeting the protein of interest without prior knowledge of its ligands. Fortunately, a wide variety of *in vitro* evolution methods are available. This includes, but is not limited to, mRNA display, yeast display, and phage display (7, 8). A common feature of these methods is the connection of a DNA- or RNA-encoded peptide to the genetic code that determines its sequence. This allows deconvolution of the results of selection by simple sequencing.

The success of any display method is determined by the design and quality of the library used. Libraries were originally composed of linear peptides, but a wide range of complex libraries including phage display of antibody fragments (9), antibodies (10), or even complete proteomes have since been developed. Small molecules remain highly desirable for certain uses, including targeting of proteins of interest using fully synthetic antibody mimetics. Therefore, displays of complex peptides cyclized though disulfide bridges or chemically modified using a variety of reagents also have been explored (11, 12, 13).

We set out to search for new ligands of prostate-specific membrane antigen (PSMA), a metalloprotease overexpressed in prostate cancer and neovasculature of solid tumors. The significance of PSMA in cancer diagnostics and treatment has been long investigated. The surface expression of PSMA makes it an ideal target for both imaging and possible therapy or even immunotherapy. Antibody-drug conjugates, radioligands, bispecific T-cell engagers, and CAR T-cells are being investigated (14, 15, 16), some in clinical trials.

For the discovery of new ligands, we set out by using a library of bicyclic peptides, with the ultimate goal of using them as target recognition moieties on iBodies. We found that conjugating a weakly binding peptide with unfavorable properties with a copolymer backbone improves the stability, solubility, and affinity. The resulting copolymer can be further decorated with fluorophores, affinity anchors, and other targeting or visualization moieties. Our findings indicate that such fully synthetic polymer conjugates can even recognize different conformations of the target protein, demonstrating their potential to replace antibodies in a variety of applications.

## Results

### Phage display identifies a bicyclic peptide ligand of PSMA

To identify new ligands of PSMA, we employed a phage display approach with chemically modified peptides. Introducing chemical modifications to linear peptides to induce a specific peptide conformation can have a significant impact on the peptide-binding properties (17). We used the previously described phage library A (18) kindly provided by C. Heinis (Ecole polytechnique fédérale de Lausanne). This fd phage library contains tetradeca- and pentadecapeptides that include three cysteine residues and a four-amino-acid linker. The cysteine residues were cross-linked by thioether bonds, yielding differently sized bicycles. Phages from library A were produced as previously described (11, 18) and chemically modified with 1,3,5-tris(bromomethyl)benzene (TBMB).

Phage library A modified with TBMB was used for three-round panning against the recombinant extracellular part of PSMA immobilized on magnetic beads. For each round of the selection, 3 μg of biotinylated PSMA was used (see Experimental procedures). The phage pool resulting from each round of selection was used to infect susceptible TG1 bacteria, amplified, and used in subsequent rounds. In the second and third rounds, the chemically modified phage pool selected in the previous round was divided in half; one half was used for panning against PSMA^+^ beads and the other was used as a control for panning against PSMA^−^ beads. The phage titers rose throughout selection, resulting in a significantly higher titer on PSMA^+^ beads than on the control PSMA^−^ beads after round three (Fig. S1), indicating selection of PSMA-specific phage clones.

Isolated DNA from the second and the third rounds was used as a template for sample preparation for next-generation sequencing. Initial PCR reactions introduced barcodes that allowed sample pooling and subsequent deconvolution of the results. PCR amplicons were sequenced using Illumina next-generation sequencing (Eurofins Genomics). The obtained reads were analyzed in Matlab as previously described (19, 20), with approximately 300,000 reads per sample after quality control and translation.

**Figure 1:**
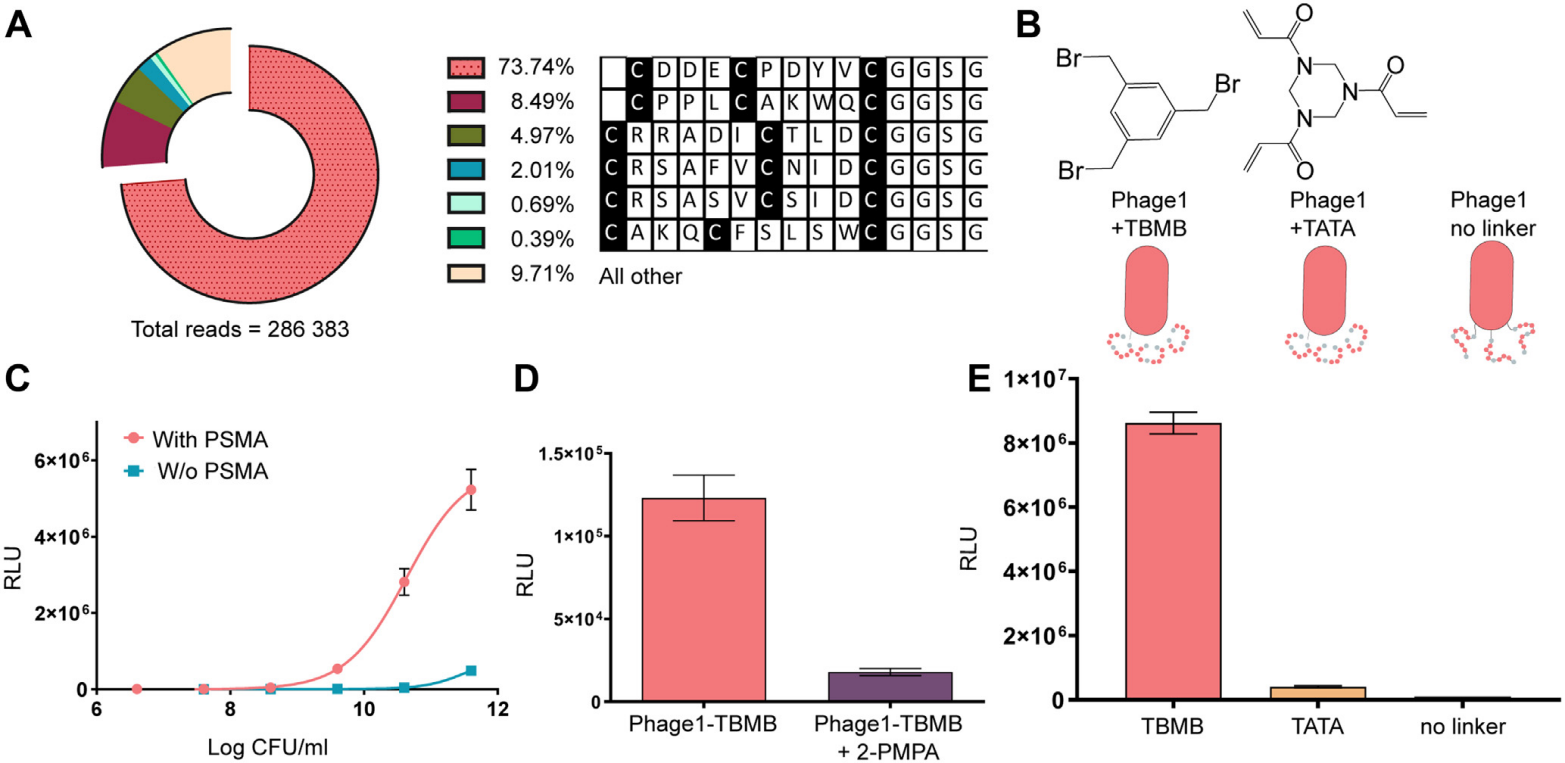
**Phage display results.***A*, next-generation sequencing revealed that 74% of the resulting phage pool was composed of one clone. *B*, the lead clone, Phage1, was isolated and cross-linked using either 1,3,5-tris(bromomethyl)benzene (TBMB) or 1,3,5-triacryloyl-1,3,5-triazinane (TATA) or left unmodified (no linker). *C*, titration of Phage1 modified with TBMB (Phage1-TBMB) on an ELISA layer with (magenta) or without (cyan) PSMA (representative of >6 experiments, n = 3). *D*, comparison of the binding of Phage1-TBMB to PSMA in the presence or absence of the inhibitor 2-PMPA (12.5 μM, n = 4). *E*, analysis of the binding of modified phages to PSMA using ELISA (representative of two experiments, n = 3).

Analysis of next-generation sequencing of library A revealed several phage clones as candidate binders of PSMA. We observed a shift in the distribution and percentage occupied by specific phage clones between rounds 2 and 3, as expected in a successful selection. After the third round of selection, a single clone, Phage1, represented approximately three-quarters of the resulting pool (Fig. 1*A*). We repeated the selection process two more times, and sequencing of the resulting pools gave very similar results (see Fig. S2), showing that the selection reproducibly leads to the identification of Phage 1 as well as other peptide motifs.

### Phage1 binds PSMA specifically and can be outcompeted by a PSMA inhibitor

To avoid lengthy preparation of a number of peptides, we first isolated Phage1 and used it for initial verification of the phage display results. We prepared three variants of the leading clone: modified with TBMB; modified with the alternative linker 1,3,5-triacryloyl-1,3,5-triazinane (TATA); and unmodified (Fig. 1*B*). Phage1 cyclized with TBMB was titrated on ELISA layers with and without PSMA (Fig. 1*C*). We observed specific binding to PSMA with a substantial signal-to-background ratio (over 60×) confirming that the selected clone indeed binds the target protein.

To further assess the specificity of the interaction, we performed a competition experiment with a known specific PSMA inhibitor, 2-(phosphonomethyl)pentane-1,5-dioic acid (2-PMPA). We detected a decrease in the signal showing that 2-PMPA competed out the binding of Phage1 (Fig. 1*D*).

To test whether the phage binds PSMA through the displayed peptide and analyze the role of the cyclization reagent used, Phage ELISA results showed that binding of PSMA by Phage1 is greatly increased by cyclization with TBMB (Fig. 1*E*).

The dependence of Phage1 binding on TBMB cyclization suggested that the specific interaction with PSMA occurs through the displayed peptide. Heinis and coworkers previously established that different cyclization linkers can induce different conformations of the modified peptides (17), which points to the importance of the peptide binder conformation for binding.

### iBody 1 binds PSMA and specifically recognizes the uninhibited enzyme

**Figure 2:**
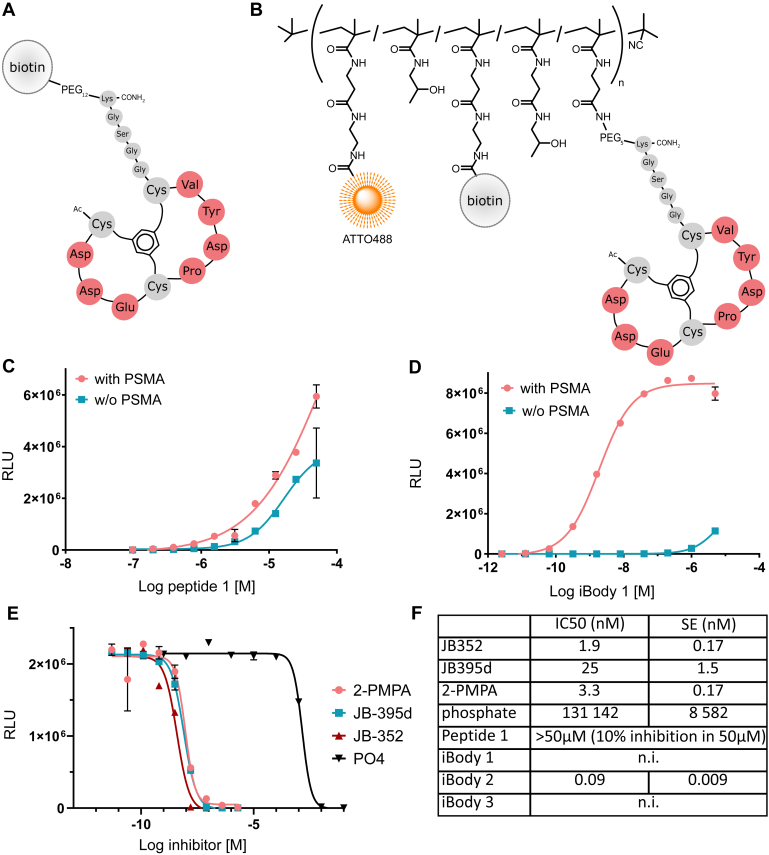
**The preparation of Peptide 1 and iBody 1 and their binding and activity on PSMA.** Schematic representations of (*A*) bicyclic Peptide 1 (compound 1) joined by a PEG linker to biotin and (*B*) the iBody 1 that also carries ATTO488 and biotin moieties and the analysis of the binding and inhibitory properties of Peptide 1 and iBody 1. In both cases the N-terminus is acetylated (Ac) and the C-terminus is amide (CONH_2_). *C*, binding of Peptide 1 (representative of five experiments, n = 3) and (*D*) iBody 1 on ELISA layers with (*coral*) or without (*cyan*) immobilized PSMA (representative of five experiments, n = 3). *E*, iBody 1 (5 nM) was outcompeted by four different PSMA inhibitors (representative of >3 experiments, n = 3). *F*, IC_50_ values of PSMA inhibitors, Peptide 1, iBody 1, iBody 2 (positive control, targeted by inhibitors), and iBody 3 (negative control, without targeting ligand) as determined by an activity assay. n.i. indicates no inhibition, standard error (SE) was calculated using GraFit software (representative of >3 experiments, n = 3).

Next, we synthesized the bicyclic peptide displayed on Phage1 (Peptide 1) and added a lysine residue after the GGSG spacer to enable simple modification. Peptide 1 was prepared using standard solid-phase synthesis followed by TBMB cyclization and modified in two different ways: by addition of a PEG_12_ linker and a biotin group (compound 1, Fig. 2*A*) or by addition of a PEG_5_ linker ending with an amine group (compound 2). The latter was used for preparation of pHPMA copolymer conjugate iBody 1 (Fig. 2*B* and Fig. S3). iBody 1 carries the bicyclic peptide (compound 2, 9.8× per polymer), the ATTO488 fluorophore (3.0× per polymer), and biotin (11.6× per polymer). These components allow easy visualization, detection, and immobilization of the target protein.

To compare the binding properties of Peptide 1 and iBody 1, we performed ELISA with immobilized PSMA. While the peptide showed rather nonspecific and weak binding (EC_50_ higher than 5 μM; Fig. 2*C*), iBody 1 bound PSMA with greater specificity and potency (EC_50_ = 2 nM, a three-order-of-magnitude difference; Fig. 2*D*). To verify that the iBody backbone did not interact with PSMA, we used iBody 3 without specific ligand to PSMA in ELISA and observed no specific binding to immobilized PSMA compared with the control layer without PSMA (Table S1). The improvement of EC_50_ of iBody 1 compared with Peptide 1, therefore, depended on the combination of Peptide 1 with iBody scaffold. We hypothesized that the improvement was due to the hydrophilic backbone of the iBody as well as an avidity effect.

### iBody 1 does not inhibit PSMA, but its binding is outcompeted by PSMA inhibitors

We next aimed to decipher the binding mode of the newly discovered peptide ligand of PSMA. The enzymatic activity of PSMA has been well described, and a number of highly potent inhibitors have been identified. We analyzed competition of iBody 1 with 2-PMPA, JB-395d (compound 1 in Šácha *et al*. (2)), JB-352 (compound 3 in Tykvart *et al*. (21)), and phosphate, which is present in standard phosphate-buffered saline (PBS) commonly used in biochemical assays (Fig. 2E, Fig. S4). Indeed, the binding of the iBody to PSMA on ELISA was outcompeted by all these inhibitors. Although the inhibitors prevented binding of the iBody 1, neither the iBody 1 nor the peptide inhibited PSMA activity (Fig. 2*F*). This is in contrast to iBody 2, used as a positive control, which is targeted to PSMA by JB-395d instead of the Peptide 1 and which does inhibit PSMA activity. iBody 3, used as a negative control, does not contain targeting ligands and does not inhibit PSMA as expected. Results, therefore, suggest that Peptide 1 likely binds outside the active site to a site that is not available upon inhibitor binding.

**Figure 3:**
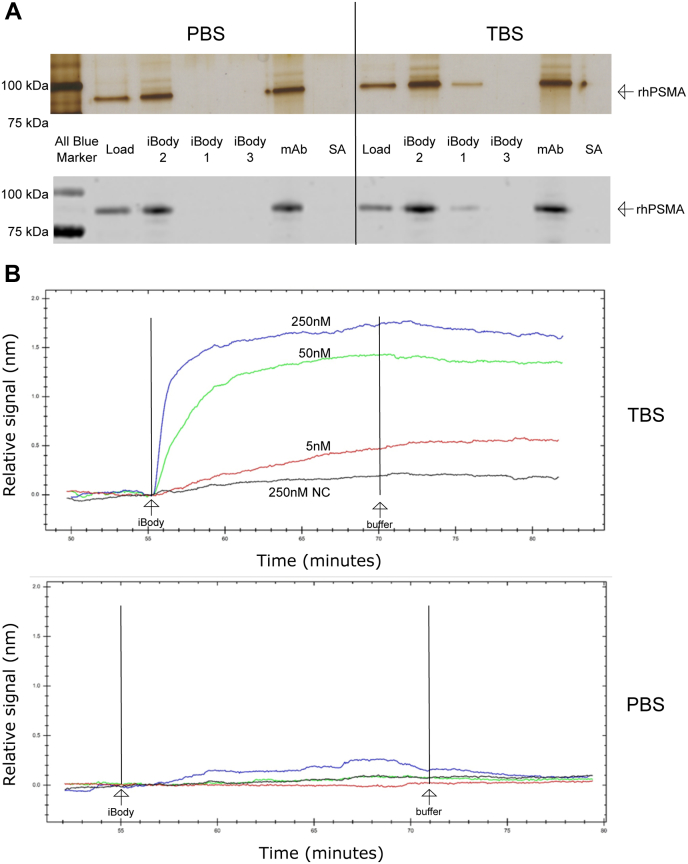
**iBody 1 binding to PSMA in solution and observation of the binding kinetics.***A*, iBody 1 binding in a pull-down assay. PSMA in either PBS or TBS was incubated with PSMA binding or control iBodies or antibodies immobilized on Streptavidin Agarose resin. Results are shown on silver-stained gels (*top*) and Western blots (*bottom*) (representative of three experiments). *B*, Kinetics of iBody 1 binding at three different concentrations to PSMA detected by surface plasmon resonance. The negative control (NC) lacks PSMA and was incubated with the highest concentration of iBody 1 used in the experiment. Binding kinetics were observed both in TBS (*top*) and PBS (*bottom*) (representative of two experiments).

We verified the hypothesis that iBody 1 binds specifically to the uninhibited conformation of PSMA using surface plasmon resonance (SPR) and pull-down assays in the presence of standard PBS, which inhibits PSMA, and Tris-buffered saline (TBS), which does not. We found that the iBody binds PSMA only in the absence of phosphate, as shown by both silver-stained gels and Western blots (Fig. 3*A*). SPR kinetics measurements revealed that virtually no binding occurred in PBS, while binding in TBS proceeded as expected (Fig. 3*B*).

**Figure 4:**
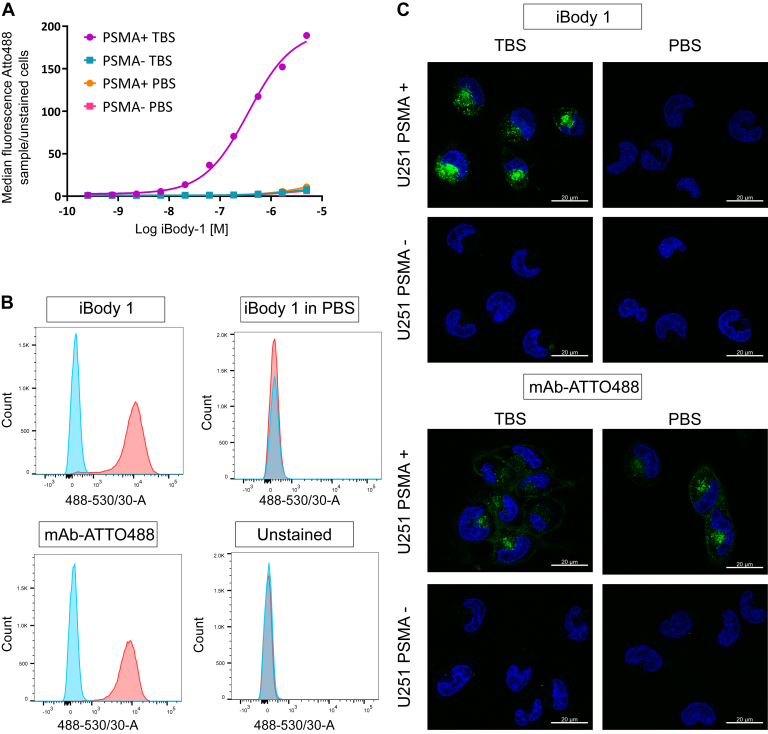
**iBody 1 binds to PSMA-expressing cells in flow cytometry and confocal microscopy experiments.***A*, U251-PSMA-tet-off cells were cultured with (PSMA^−^) or without (PSMA^+^) doxycycline, trypsinized, counted, and incubated with a titration series of iBody 1 in either PBS or TBS. Cells were washed once and samples were measured on a BD LSRFortessa flow cytometer. Median fluorescence of ATTO488 normalized to unstained cells is shown (representative of >3 experiments). *B*, histograms show the distribution of the fluorescence intensity in cell samples treated with iBody 1 (555 nM), stained or unstained with the PSMA-specific monoclonal antibody (mAb) 2G7 conjugated with ATTO488 and 555 nM iBody 1 incubated with the cells in PBS. PSMA^+^ (*red*) and PSMA^−^ (*blue*) U251 cells are shown for each condition. *C*, confocal microscopy showing distribution of iBody 1 in cells after a 30-min incubation with PSMA^+^ or PSMA^−^ cells in TBS or PBS; mAb 2G7-ATTO488 antibody is used as a positive control (ATTO488 – *green channel*, Hoechst 34580 – *blue channel*) (representative of three experiments).

To verify that this selectivity for the uninhibited form of PSMA is not an artifact of the recombinant protein used in the biochemical assays, we conducted flow cytometry experiments with the U251 clonal cell line transfected with full-length human PSMA under a tet-off promotor (4). Titration assays revealed that in TBS iBody 1 specifically binds to PSMA^+^ cells, while no binding is observed in PBS, KH_2_PO_4_, 2-PMPA, or on control PSMA^−^ cells (Fig. 4, *A* and *B*, Figs. S5 and S6). These findings show that the binding is not dependent on the PSMA expression system, the construct used, or different glycosylation patterns on the protein, but rather on inhibition of PSMA.

To determine the distribution of the iBody 1 inside PSMA^+^ cells, we used confocal microscopy imaging. We observed that the iBody 1 binds to PSMA-expressing cells in TBS but not PBS. When binding occurs in TBS, the iBody 1 internalizes, with almost all bound iBody 1 seen inside the cytoplasm after a 30-min incubation at 37 °C (Fig. 4*C*). In contrast, anti-PSMA antibody 2G7 (22) binds to PSMA-expressing cells regardless of the buffer used. This indicates that iBody 1 senses the conformational state of PSMA in the cells, while the antibody binding is insensitive to the presence of inhibitors.

In both flow cytometry and confocal microscopy experiments, the binding of iBody 1 to PSMA^+^ cells is highly dependent on the presence of phosphate (Fig. 4), which is consistent with the results of biochemical methods with a truncated version of the target enzyme.

## Discussion

Methods for *in vitro* selection of protein ligands often yield poorly soluble hydrophobic compounds with binding constants in the micromolar range. Synthetic peptides typically lose some specificity and binding sensitivity compared with the corresponding phage ancestor. Here, we present data from a typical phage display selection of a peptide ligand of PSMA. While the selected bicyclic peptide showed promising binding, it was not sufficiently selective or potent as a synthetic small molecule ligand. Published bicyclic peptide binders vary significantly in their binding affinity (23). The number of bicyclic peptides discarded from further investigation due to insufficient affinity is not known, however, but probably causes that a number of peptides are not utilized.

However, in this instance, combining the peptide with a polymer-based hydrophilic carrier led to significant improvements; the iBody 1 exhibited a nearly three-order-of-magnitude increase in binding over the peptide alone. The presence of several peptides covalently attached to one iBody likely better mimics the situation on the phage P3 protein. Furthermore, attachment to a hydrophilic and easy-to-modify polymer scaffold can greatly improve the solubility of the ligand and enable further modification by addition of affinity molecules, fluorophores, or other ligands. This strategy circumvents a common problem in phage display selection—identification of a promising lead phage clone followed by insufficient affinity and selectivity of the encoded peptide. Combining a small molecule with unfavorable properties with a large hydrophilic copolymer-based scaffold offers a new approach to translate hits from *in vitro* selection methods to biochemical applications.

Importantly, we observed that while the bicyclic peptide itself does not inhibit PSMA, it also does not recognize the inhibited form of the enzyme. Crystal structures of PSMA established that when small-molecule inhibitors such as 2-PMPA (24) or JB-352 bind to the enzyme, the active site adopts a closed conformation. In contrast, inhibitors with a PEG linker attached to the zinc-binding “warhead” of the compound, such as JB-395d, keep the so-called “entrance lid” of the active site in an open position. This suggests that other structural changes occur upon inhibitor binding to the PSMA active site, resulting in unavailability of the binding site for the bicyclic peptide. We conclude that Peptide 1 and the corresponding iBody 1 likely bind outside of the active site to an exosite that is only available on the uninhibited enzyme.

This study presents an antibody mimetic able to bind selectively to a specific conformation of the target protein. This proof of principle shows that antibody mimetics can achieve conformation selectivity, similar to some antibodies (10) and paves the way for development of versatile, stable, simple-to-prepare synthetic antibody mimetics that recognize functionally and structurally defined epitopes.

## Experimental procedures

### Bicyclic phage display

Phage display using bicyclic peptide phage libraries kindly supplied by C. Heinis (EPFL) was performed as previously described (18). Briefly, 3 μg Avi-PSMA previously expressed in our laboratory (labeled Avi-GCPII in Tykvart *et al*. (25)) was incubated with 30 μl of a 50% slurry of M-280 Streptavidin Dynabeads (Invitrogen) in the first and third round of selection. For the second round of selection, we used 20 μl of NeutrAvidine beads prepared through conjugation of NeutrAvidine (Thermo Fisher Scientific) to Dynabeads M-280 Tosylactivated (Invitrogen) according to the manufacturer's protocol. Phage library A (or in subsequent rounds the phage pool from the previous round of display) was blocked (1% BSA) and incubated with magnetic beads with PSMA (control panning with beads without PSMA was performed in second and third round). Beads were washed six times with buffer with detergent (0.1% Tween-20), transferred to fresh tubes, and washed three times without detergent. Phages were eluted using 50 mM glycine buffer (pH 2.2) for 5 min, neutralized by 1 M Tris (pH 8.0), and infected into susceptible TG1 bacteria. Samples were taken to evaluate the library preparation as well as the resulting phage pools after the display round itself.

### Next-generation sequencing

A 300-μl aliquot from a total of 7.5 ml TG1 bacteria infected with the resulting phage pool after the second and third round of selection was used for plasmid DNA isolation using the Zyppy Plasmid Miniprep Kit (ZYMO RESEARCH). The resulting DNA (100 pg) was amplified in a PCR reaction using Phusion HF polymerase (NEB) in a 50-μl reaction volume. The amplified DNA fragment was separated on a 2% UltraPure agarose (Thermo Fisher Scientific) gel. The fragment around 150 bp was excised, and DNA was extracted using the QIAEX II Gel Extraction Kit (QIAGEN). DNA was quantified using Nanodrop and the Qubit dsDNA HS kit (Thermo Fisher Scientific). Qubit values were used to pool samples equivalently, and the resulting mix was sent for paired-end 125 bp Illumina next-generation sequencing at Eurofins Genomics (then GATC).

### Analysis of next-generation sequencing results

Previously published Matlab scripts (19) were used for analysis of results from next-generation sequencing.

### Phage clone isolation

After the final round of selection, ten colonies of TG1 bacteria infected with the resulting phage pool were picked. DNA was isolated using the Zyppy Plasmid Miniprep Kit (ZYMO RESEARCH) and Sanger sequenced by Eurofins Genomics. The dominant phage clone was identified in several colonies, and one was grown to create a frozen bacterial stock in 20% glycerol. The phage clone was grown from this stock, isolated, divided into three parts, and chemically modified using the same procedure as for the phage libraries. The nonmodified sample was kept in equivalent conditions without the addition of TBMB or TATA.

### Phage titration assay

All phage samples were prepared in ten times dilution series and 20 μl from each step was transferred to 180 μl of TG1 bacteria (previously grown to OD_600_ 0.4). After infection in 37 °C for 90 min, 10 μl from each sample was spotted on chloramphenicol agar plates and grown overnight in 37 °C. Since phages carry resistance to chloramphenicol, the infected bacteria gain this resistance, while the uninfected ones do not. Colonies were counted and the original number of phages in the given sample was calculated based on the dilution step.

### Phage ELISA

A black flat-bottom 96-well MaxiSorb plate (Nunc) was coated with 100 μl 2G7 antibody (22) (0.5 μg/well in TBS), incubated for 1 h at RT, and blocked with 200 μl 1% BSA in TBS at 4 °C overnight. After three washes with 200 μl TBST′, the wells were incubated with the biotinylated recombinant extracellular part of PSMA (25) (100 ng/well, 1 h at RT with shaking) or buffer for control wells. After three additional washes, the dilution series of a given phage clone in 1% BSA TBST′ was added (2 h at RT with shaking) and again washed thrice. For competition ELISA, 25 μM 2-PMPA was added in a volume of 50 μl and incubated for 30 min with shaking. Phage1 modified with TBMB (2.8 x 10 (9) cfu/ml as determined by titration assay in a volume of 50 μl) was added to each well and incubated for 1 h at RT with shaking. Wells were then incubated with HRP-conjugated a-M13 phage antibody (GE Healthcare) (1:1000, 2% BSA, TBST′). Following four washes with TBST′, chemiluminescence enhanced by 4-iodophenol was measured on an Infinite M1000 plate reader (Tecan). Readout is measured using chemiluminescent substrate (2) and shown as relative light units (RLU).

### Peptide synthesis

Ac-CDDECPDYVCGGSGK-NH_2_ was synthesized by SPPS on a Liberty Blue peptide synthesizer (CEM, USA) using standard Fmoc chemistry protocols, DIC/Oxyma-Pure coupling reagents and Rink amide MBHA resin support (0.2 mmol scale, 10 equiv. amino acid excess). Fmoc groups were removed with 20% piperidine. Peptide amino acids side chains were deprotected, and peptide was cleaved off the resin with a mixture of TFA/EDT/anisole/thioanisole/water (90:2.5:2.5:2.5:2.5) for 1.5 h at RT.

The cleaved peptide was lyophilized and purified by RP HPLC (Vydac 218TP101522 column) using methanol and water with 0.05% TFA as solvents. The purity was assessed by analytical RP-HPLC (Vydac 218TP54 column) and LC/MS (Agilent Technologies 6230 ToF LC/MS). The mass was confirmed by MALDI-ToF MS: Ac-CDDECPDYVCGGSGK-NH_2_ [M + H]+ 1588.6.

### Peptide cyclization

Pure peptide was dissolved in 80% aqueous buffer (20 mM NH_4_HCO_3_, 5 mM EDTA, pH 8.0) and 20% acetonitrile. TBMB (1.5 equiv.) dissolved in acetonitrile was added, and the compounds were mixed overnight at RT. The reaction mixture was lyophilized. The modified peptide was purified by RP HPLC, and purity was assessed as described above. The mass was confirmed by MALDI-ToF MS: 1,3,5-tris(bromomethyl)benzene(TBMB)-cyclized Ac-CDDECPDYVCGGSGK-NH_2_ [M + H]+ 1702.7.

### Synthesis of PEGylated cyclic peptides

Biotin-NH-(CH_2_CH_2_O)_12_-CH_2_CH_2_-COOH or Boc-NH-(CH_2_CH_2_O)_5_-CH_2_CH_2_-COOH was activated with 1 equiv. HOSu and 5 equiv. DIC in DMF for 2 h. Solvent and excess DIC were removed on a vacuum oil pump overnight. Bicyclic peptide was incubated in DMF with three equiv. of PEG compound-OSu ester in the presence of three equiv. DIPEA overnight.

Reaction mixtures were evaporated. The PEGylated cyclic peptides were purified by RP HPLC, and purity was assessed as described above:

Compound 1, TBMB-cyclized Ac-CDDECPDYVCGGSGK[(CO)CH_2_CH_2_(OCH_2_CH_2_)_12_-NH-Biotin]-NH_2_ [M + H]+ 2529.0
TBMB-cyclized Ac-CDDECPDYVCGGSGK[(CO)CH_2_CH_2_(OCH_2_CH_2_)_5_-NH-Boc]-NH_2_ [M + H]+ 2093.6

The Boc group was removed by incubation with TFA for 0.5 h, and the compound was lyophilized. The purity was assessed as described above.

Compound 2, TBMB-cyclized Ac-CDDECPDYVCGGSGK[(CO)CH_2_CH_2_(OCH_2_CH_2_)_5_-NH_2_]-NH_2_ [M + H]+ 1993.6

### Synthesis of monomers, polymer precursors, and polymer conjugates

The monomers *N*-(2-hydroxypropyl)methacrylamide (HPMA) and 3-(3-methacrylamido-propanoyl)thiazolidine-2-thione (Ma-ß-Ala-TT) were synthesized as previously described (26), as was the chain transfer agent S-2-cyano-2-propyl S′-ethyl trithiocarbonate (27). The polymer precursor poly(HPMA-co-Ma-ß-Ala-TT) was prepared by reversible addition-fragmentation chain transfer (RAFT) copolymerization (28). Ma-ß-Ala-TT (308 mg, 1.19 mmol) was dissolved in 2.8 ml *N*,*N*-dimethylacetamide (DMAA), and the solution was diluted with *tert*-butanol (11.4 ml). HPMA (1.25 g, 8.73 mmol), S-2-cyano-2-propyl S′-ethyl trithiocarbonate (3.13 mg, 1.52 ×10^−2^ mmol), and the initiator 2,2′-azobis(4-methoxy-2,4-dimethylvaleronitrile) (V-70; Wako Chemicals GmbH) (2.35 mg, 7.63 × 10^–3^ mmol) were added, and the solution was introduced into a polymerization ampule. The mixture was bubbled with argon for 10 min, and the ampule was sealed. Polymerization was carried out at 40 °C for 16 h. The polymer precursor was isolated by precipitation into a mixture of acetone: diethyl ether (3:1, 250 ml), filtered, washed with acetone (20 ml) and diethyl ether (20 ml), and dried *in vacuo*. The yield of polymerization was 0.718 g (46%). The terminating trithiocarbonate group was removed as described by Perrier (29). This yielded the polymer precursor poly(HPMA-co-Ma-β-Ala-TT) with molecular weight *M*_n_ = 63,000 g/mol, *M*_w_ = 70,600 g/mol, dispersity *Ð* = 1.12, and content of reactive thiazolidine-2-thione groups 10.9 mol%.

### Synthesis of iBody 1

The polymer precursor poly(HPMA-*co*-Ma-β-Ala-TT) (6.8 mg; *M*_n_ = 63,000 g/mol; *M*_w_ = 70,600 g/mol; Ð = 1.12; 10.9 mol% TT, 4.75 μmol reactive groups), compound 2 (2.4 mg, 1.15 μmol), *N*-(2-aminoethyl)biotinamide hydrobromide (biotin-ED-NH_2_; 0.7 mg, 1.9 μmol), and ATTO488-NH_2_ (0.23 mg, 0.27 μmol) were dissolved in 0.3 ml DMSO. Then, 2.3 μl (3.3 μmol) of *N*,*N*-diisopropylethylamine (DIPEA) was added. The reaction was carried out for 4.5 h at room temperature, 2 μl of 1-aminopropan-2-ol was added, and the reaction was stirred for an additional 10 min. A solution of the polymer conjugate poly(HPMA-*co*-Ma-β-Ala-compound 2-*co*-Ma-β-Ala-ATTO488-*co*-Ma-β-Ala-NH-ED-biotin) was diluted with 1.5 ml methanol and purified on a Sephadex LH-20 chromatography column in methanol. Methanol was evaporated, and the polymer conjugate was dissolved in 1.5 ml distilled water, purified on a Sephadex G-25 chromatography column, and lyophilized. The yield of iBody 1 was 8.0 mg. (M_n_ = 88,100 g/mol, M_w_ = 104,000 g/mol, Ð = 1.18). The content of compound 2 was 20.4 wt%, the biotin content was 3.5 wt%, and the ATTO488 content was 2.0 wt%.

### Determination of the molecular weight and composition of the HPMA conjugate iBody 1

The weight-average molecular weights (*M*_w_), number average molecular weights (*M*_n_), and dispersities (*Ð*) of the polymer precursor and conjugate were determined using a Shimadzu HPLC system equipped with a UV detector, an OptilabrEX differential refractometer, a DAWN 8 multiangle light scattering detector (Wyatt Technology), and a TSKgel G4000SWXL size-exclusion chromatography column. The *M*_w_, *M*_n_ and *Đ* were calculated using Astra V software. The refractive index increment dn/dc = 0.167 ml/g was used for calculation. These experiments were conducted in buffer containing 300 mM sodium acetate, pH 6.5, and methanol (20%/80% v/v). The flow rate was 0.5 ml/min. The content of TT reactive groups in the polymer precursor was determined spectrophotometrically (ε_302nm_ = 10,600 L mol^−1^ cm^−1^, methanol). The content of the fluorophore ATTO488 in the HPMA conjugate was determined using spectrophotometry (ε_502nm_ = 90,000 L mol^−1^ cm^−1^, water). The content of biotin in the HPMA conjugate was determined using the HABA/Avidin Reagent kit (Sigma-Aldrich) for spectrophotometric determination at 500 nm according to the manufacturer's instructions (Sigma, H 2135); the results were corrected for the effect of ATTO488 absorbance at 500 nm.

The content of the targeting ligand in the conjugate was determined by amino acid analysis with precolumn 2,3-naphthalenedicarboxaldehyde (NDA)/NaCN derivatization on a Shimadzu HPLC system with an RF-20A fluorescence detector (Ex = 229 nm/Em = 490 nm) using a reverse-phase Chromolith RP18e column (100 x 4.6 mm). A gradient of solvent sodium acetate buffer in methanol was used with a flow rate of 1 ml/min. Prior to analysis, the sample was hydrolyzed with 6 M HCl at 115 °C for 16 h. The hydrolysate was dried and dissolved in water.

iBodies 2 (poly(HPMA-*co*-Ma-β-Ala-(JB395d)-*co*-Ma-β-Ala-ATTO488-*co*-Ma-β-Ala-NH-ED-biotin)) and 3 (poly(HPMA-*co*-Ma-β-Ala-ATTO488-*co*-Ma-β-Ala-NH-ED-biotin)) were previously described (iBody 2 corresponds to iBody 1 in Šácha *et al*. (2), iBody 3 corresponds to iBody 5 in Šácha *et al*., (2)). Molar solutions of iBodies were prepared using Mw′ values as previously described (6).

### Labeling of anti-PSMA monoclonal antibody 2G7 with ATTO488

A 235-μL aliquot of 2G7 antibody (22) (0.69 mg/ml) was transferred into PBS (pH 8.0) by two rounds of centrifugation using an Amicon ultra-0.5 Centrifugal Filter (Millipore) with a 30 kDa molecular weight cutoff (5 min, 14,000*g*). Each time, the antibody solution was resuspended into a 500 μl volume with PBS (pH 8.0), and the final antibody solution was adjusted to 300 μl. Next, 1 μl of 10 mM ATTO488-NHS ester (ATTO-TEC) in anhydrous DMSO was added, mixed by three short vortex pulses, and incubated at RT on a rotating wheel for 60 min. Finally, excess ATTO488-NHS ester was removed by two rounds of purification using an Amicon ultra-0.5 Centrifugal Filter with a 30 kDa molecular weight cutoff (5 min, 14,000*g*) and stored at 4 °C.

### ELISA

A black flat-bottom 96-well MaxiSorb plate (Nunc) was coated with 100 μl 2G7 antibody (22) in TBS (0.5 μg/well), incubated for 1 h at RT, and blocked with 200 μl 1% BSA in TBS at 4 °C overnight. After three washes with 200 μl TBST′, the wells were incubated with the recombinant extracellular part of PSMA (50 ng/well, 1 h at RT with shaking) or buffer (control). After three washes, dilution series of iBody or peptide in TBST′ were added (90 min at RT with shaking), and wells were again washed thrice. For competition ELISA, dilution series of PSMA inhibitors were added (50 μl/well) and incubated for 30 min with shaking. Then 20 nM iBody in a 50 μl volume was added to each well and incubated for another 60 min at RT with shaking. Finally, the wells were incubated with HRP-conjugated NeutrAvidine (10 ng/well, TBST′). After six washes with TBST′, chemiluminescence enhanced by 4-iodophenol was measured on an Infinite M1000 plate reader (Tecan). Values are shown as relative light units (RLU).

### PSMA activity assay

PSMA activity assays were performed as previously described (21). Briefly, the activity of AviPSMA was measured by 20-min incubation of the enzyme with substrate pteroyl-di-L-glutamate (Schircks Laboratories) and a titration of indicated inhibitors (37 °C). Reactions were stopped using 25 μM 2-PMPA, cooled to 4 °C and measured on Agilent 1200 Series. IC_50_ values were calculated using GraFit v.5.0.11 (Erithacus Software Ltd).

### Immunoprecipitation of PSMA

Pull-downs of PSMA protein from spiked buffer were performed either in PBS or TBS. iBody 1, iBody 2 (positive control targeted by PSMA inhibitor), iBody 3 (negative control lacking a targeting ligand), and biotinylated anti-GCPII antibody 2G7 (22) were first immobilized on functionalized beads. Streptavidin Agarose Ultra Performance resin (Solulink) was used to immobilize the molecules *via* their biotin ligands by incubating 200 μl of each 150 nM species with 30 μl of pure resin for 90 min at RT. Resins were then washed three times with 200 μl buffer containing detergent (0.1% Tween 20). Each immobilized species was then mixed with 200 μl buffer containing 1 μg PSMA (labeled rhGCPII in Barinka *et al*. (30)), allowed to incubate for 60 min at RT, and subsequently washed five times. Finally, the immunoprecipitated species were eluted from the resin by incubation in 30 μl SDS buffer for 10 min at 98 °C. Samples (5 μl of each) were loaded and separated on a 12% polyacrylamide gel (SDS-PAGE, 220 V, 60 min, 4 °C). Standard silver staining and Western blotting procedures were used to visualize the immunoprecipitated proteins.

### SPR

Gold-coated glass-based SPR chips were functionalized with alkanethiol molecules carrying terminal carboxylic groups by a mix of HS-C_11_-(EG)_6_-OCH_2_-COOH and HS-(CH_2_)_11_-(CH_2_CH_2_O)_6_-OCH_2_-COOH and HS-(CH_2_)_11_-(CH_2_CH_2_O)_4_-OH HS-C_11_-(EG)_4_-OH (3/7 ratio of -COOH/-OH; final concentration 200 μM). Carboxylic groups were then activated by treating the functionalized chip with EDC/NHS (1-ethyl-3-(3-dimethylaminopropyl)carbodiimide/*N*-hydroxysuccinimide), providing the means for attachment of the anti-PSMA antibody 2G7 *via* its primary amine groups. To prevent nonspecific binding and block unreacted carboxyl groups, the chip was then treated with high-salt concentration PBS (0.5 M NaCl) and 1 M ethanolamine. Next, the recombinant extracellular domain of PSMA was bound to the 2G7 antibody in all channels but one (negative control channel). In the last binding step, the sensor chip was treated with iBody 1 in three different concentrations (red and green channels, 250 nM; blue channel, 50 nM; yellow channel, 5 nM). Finally, buffer was run through all four channels to observe the tightness of binding. This analysis was performed twice, each time under different buffer conditions (in either PBS or TBS).

### Cytometry

PSMA-transfected clonal U251 cell line was grown in full DMEM medium (Thermo Fisher, 10% FBS, 4 mM L-glutamine) with or without 100 nM doxycycline. On the day of the experiment, cells were trypsinized (2 ml trypsin/EDTA per 10 cm dish), and corresponding medium was added to stop trypsinization. Cells were centrifuged for 5 min at 200×*g*, resuspended in full medium, and kept in a thermostat for 30 min. Cells were then centrifuged, and the final pellet was resuspended in 1 ml TBS/PBS/medium. Cells were counted using a Countess Automated Cell Counter (Invitrogen) and adjusted to a concentration of 2.2 × 10^6^ cells/ml. Cell viability was at least 93%. A 90-μl aliquot from each cell suspension was added to wells in a 96-well U-shaped plate, and 10 μl of TBS containing a dilution series of peptide, iBody, or antibody was added (TBS alone for controls). After a 1-h incubation at 37 °C in a thermostat, 100 μl of corresponding buffer was added to mix the cells, cells were centrifuged (200*g*, 5 min), washed with 200 μl corresponding buffer, centrifuged again, resuspended in 150 μl, and measured on a BD LSRFortessa Flow Cytometer with HTS module. Results were analyzed using FlowJo 10 (Figs. 4 and S5, and S6).

### Confocal microscopy

PSMA-transfected clonal U251 cell line (4) was grown in full DMEM medium (Thermo Fisher, 10% FBS, 4 mM L-glutamine) with or without 0.1 ng/μl doxycycline in 4-Chamber 35 mm Glass Bottom Dishes (*In Vitro* Scientific). Cells were washed twice with TBS or PBS and incubated with various concentrations of compound for 15 min in a thermostat, followed by addition of Hoechst 34580 (Sigma) and another 15-min incubation (37 °C). After that, cells were washed and imaged using a Zeiss LSM 780 confocal microscope with an oil-immersion Plan-Apochromat 63×/1.40 Oil DIC M27 objective. Cells were imaged at RT with a 488 nm laser (max 25 mW) set to 1% (detector gain 900 V). Emission was collected from 499 to 569 nm for ATTO488, and with a 405 laser (max 30 mW) set to 1% (detector gain 900 V) emission was collected from 410 to 501 nm for Hoechst 34580 and pinhole 1 AU. All images were taken with the same setup and analyzed using ZEN 2.3 software (Carl Zeiss Microscopy).

### Data availability

No new code was generated over the course of the study. Datasets from next-generation sequencing are available upon request from the Lead Contact. Reagents generated are available upon request form the Lead Contact after a completed Material Transfer Agreement.

## Supporting information

This article contains supporting information (2, 21).

## Conflict of interest

iBody technology is protected by patents US10114014 (B2) and US10302632 (B2).
