# Supplementary material for: The development of a high-affinity conformation-sensitive antibody mimetic using a biocompatible copolymer carrier (iBody)
Source: J Biol Chem. 2021 Oct 25;297(5):101342. doi: 10.1016/j.jbc.2021.101342 (PMC8600089; doi:10.1016/j.jbc.2021.101342)
Supplement: Figures S1–S6 and Table S1 [file mmc1.pdf]

Supporting Information for

The Development of a High-Affinity Conformation-Sensitive Antibody Mimetic Using a Biocompatible Copolymer Carrier (iBody)

Blažková K.[a],[b], Beranová J.[a],[c], Hradilek M.[a], Kostka L.[d], Šubr V.[d], Etrych T. [d], Šácha P.*[a] and Konvalinka J. *[a],[b]

Contents:

Supplemental figure S1: Changes in phage titre of the phage pool after each round of selection.

Supplemental figure S2: The comparison of lead peptides from repeated selection using library A cyclized by TBMB panned against PSMA

Supplemental figure S3: Gel permeation chromatography (GPC) chromatogram of polymer precursor and polymer conjugate iBody 1.

Supplemental figure S4: Structures of PSMA inhibitors JB-395d, JB-352 and 2-PMPA.

Supplemental figure S5: Gating used for flow cytometry experiments

Supplemental figure S6: Competition of small molecular inhibitors 2-PMPA and phosphate with the binding of iBody 1 on cells expressing PSMA and control cells.

Supplemental table ST1: Comparison of specific binding of iBody 1 to PSMA or control layer (without PSMA) on ELISA compared to iBody 3 (not targeted to PSMA).

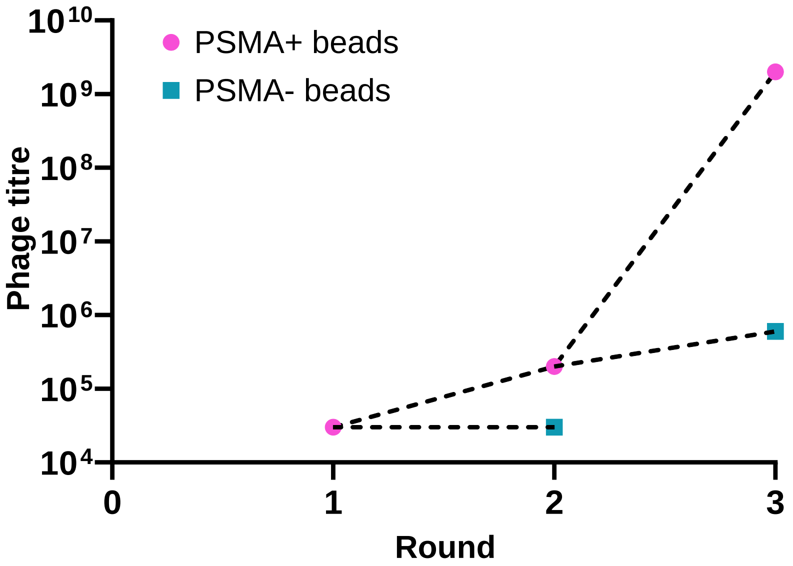

Supplemental figure S1: Changes in phage titre of the phage pool after each round of selection. Samples from each pool from positive selection or control selection were analyzed using titration assay and determined phage titres were plotted. The increase in phage titre in selection done on PSMA+ beads over control PSMA- beads correlates to the amplification of binding clone.

MAACDDECPDYVCGGSG	258006	MAACDDECPDYVCGGSG	144664	MAACDDECPDYVCGGSG	247048
MAACPPLCAKWQCGGSG	25096	MAACHRQCFSLSWCGGSG	57642	MAACHRQCFSLSWCGGSG	7928
MAACRRADICTLDCGGSG	13698	MAACRRADICTLDCGGSG	19303	MAACRRADICTLDCGGSG	6023
MAACRSAFVCNIDCGGSG	5461	MAACPPLCAKWQCGGSG	8254	MAACVLKCVGDNDCGCSG	2951
MAACRSASVCSIDCGGSG	1846	MAACVTWYCSSNYCGGSG	1692	MAACAKQCFSLSWCGGSG	1282
MAACAKQCFSLSWCGGSG	922	MAACQYAYDCDLPCGGSG	683	MAACSHTCIMHSHCGGSG	859
MAACHRQCFSLSWCGGSG	556	MAACRSAAVCNLPCGGSG	529	MAACRSAAVCNLPCGGSG	728
MAAWLEQLRGDHSFGGSG	141	MAACPYIECLYQPCGGSG	181	MAACHAHCLHSIPCGGSG	726
MAACHQHPCIHMCGGSG	92	MAACYQLCWWGWCGGSG	177	MAACDHRCHKHIQCGGSG	598
MAACHSQFHCEPRCGGSG	68	MAACDDECFSLSWCGGSG	121	MAACRRHCSMHTCGGSG	478
Total reads	333886	Total reads	255629	Total reads	294511

Supplemental figure S2: The comparison of lead peptides from repeated selection using library A cyclized by TBMB panned against PSMA. Peptides with identical or highly similar motifs are highlighted. Top 10 most abundant sequences after translation and quality control (fixerr function=10) are shown from each selection. Peptides with HPQ binding motif (Streptavidine binders), peptides with even number of cysteins and peptides without GGSG linker are not shown.

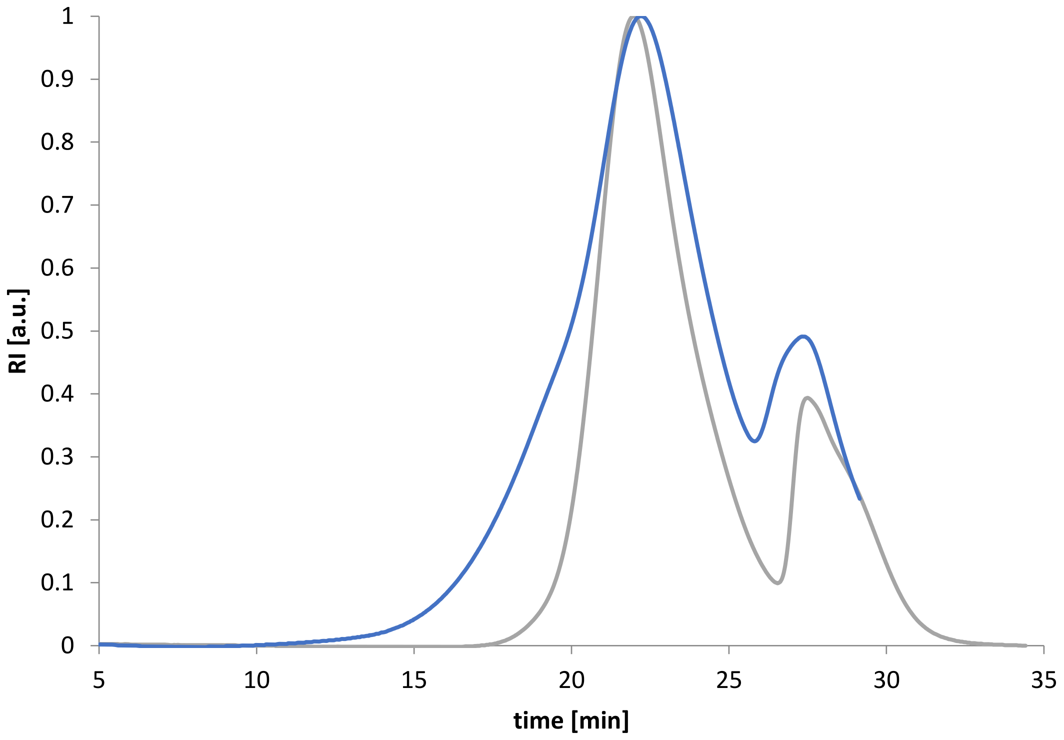

Supplemental figure S3. Gel permeation chromatography (GPC) chromatogram of polymer precursor (grey) and polymer conjugate iBody 1 (blue). Curves were obtained from dRI detector.

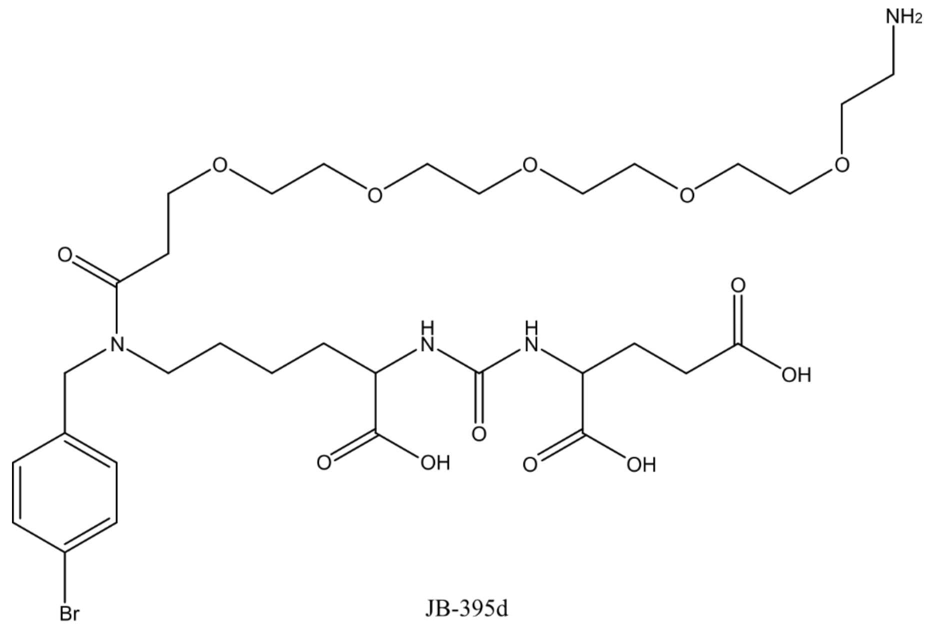

JB-395d

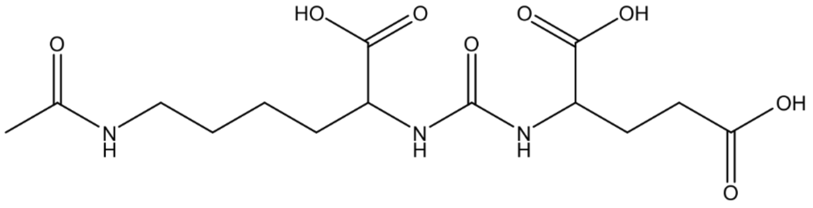

JB-352

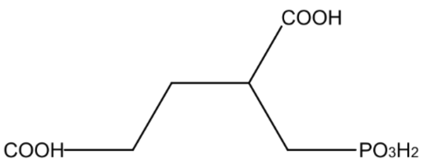

2-PMPA

Supplemental figure S4. Structures of PSMA inhibitors JB-395d (compound 1 in Šácha et al., 2016), JB-352 (compound 3 in Tykvart et al., 2014) and 2-PMPA.

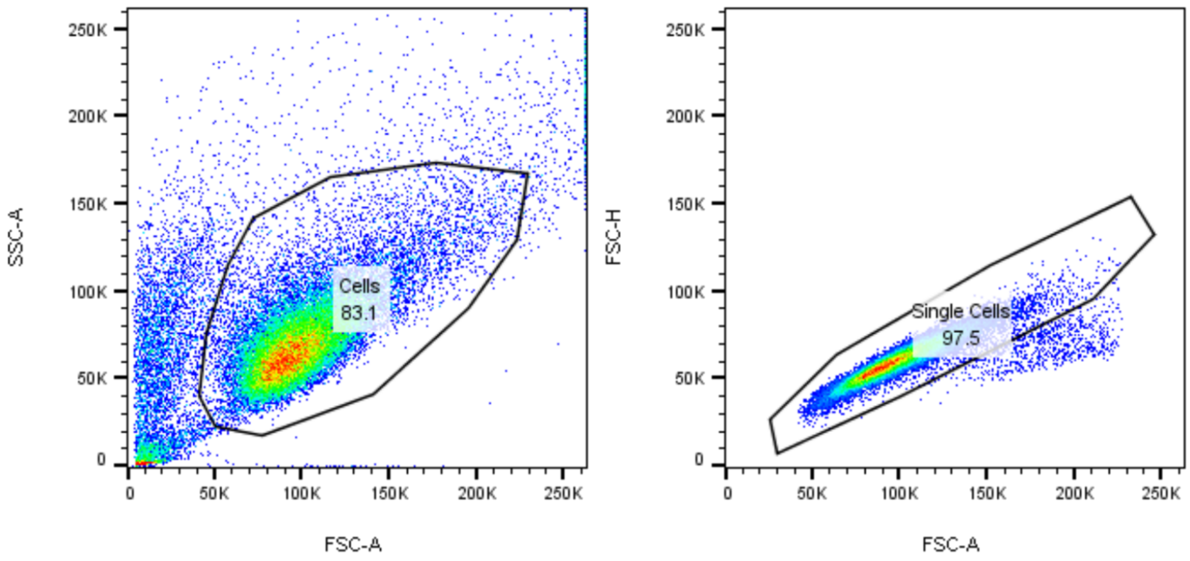

Supplemental figure S5. Gating used for flow cytometry experiments depicted in Figure 5 A) and B). Analogous gating was used for Supplemental figure S6. Main cell population was gated first, followed by gating for single cells.

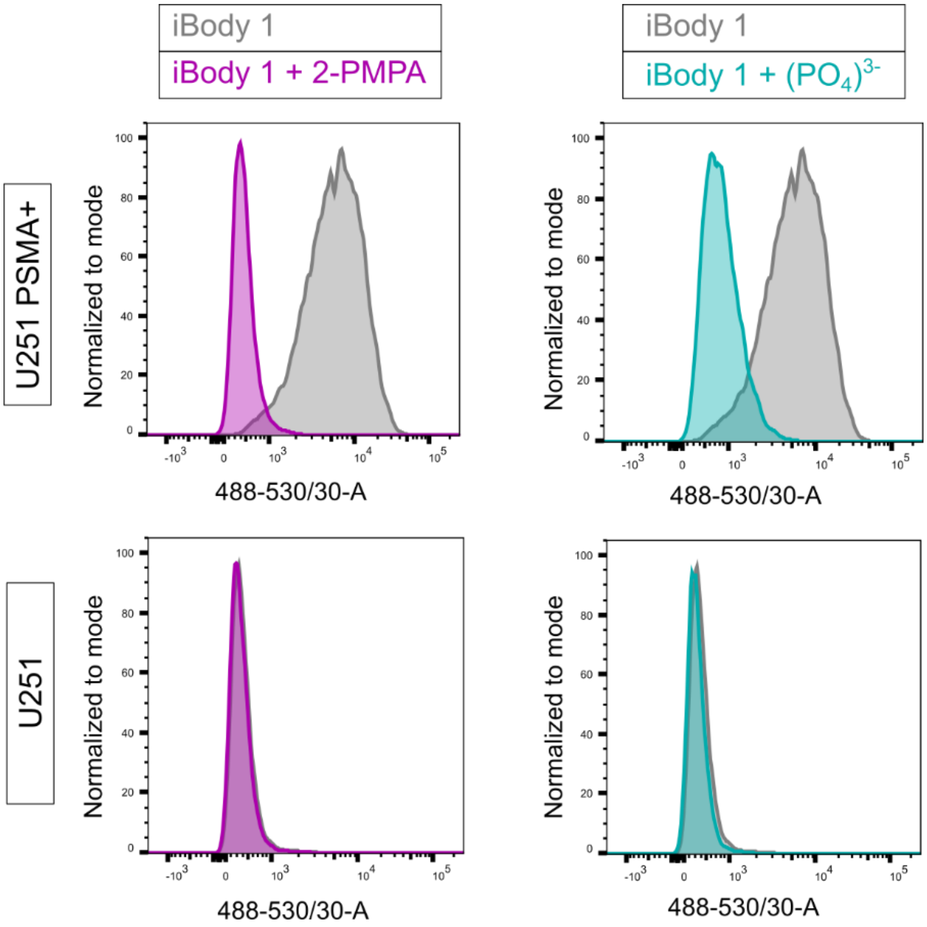

Supplemental figure S6: Competition of small molecular inhibitors 2-PMPA and phosphate with the binding of iBody 1 on cells expressing PSMA and control cells. U251 PSMA-transfected cells (PSMA+) and control untransfected U251 cells were harvested and incubated in TBS with 500 nM iBody 1 and with either 500 nM 2-PMPA (purple), 5mM KH_2PO_4 (turquoise) or TBS (positive control, grey).

Supplemental table ST1: Comparison of specific binding of iBody 1 (targeted to PSMA through the bicyclic peptides) to PSMA or control layer (without PSMA) on ELISA compared to iBody 3 (not targeted to PSMA). iBody 1 titration was done in duplicates, while iBody 3 titration was done in triplicate. Chemiluminescence was measured and values obtained for individual points were averaged and are shown in ST1 below in relative light units (RLU). Ratio was calculated for each concentration point of each iBody as value on layer with PSMA divided by the value for the same sample incubated on the layer lacking PSMA.

	concentration of iBody 1 (nM)				
	5000	1000	200	40	8
with PSMA	6373850	5914050	5048350	4164800	2978900
w/o PSMA	5500250	2193900	493730	84709	11632
Ratio:	1.2	2.7	10.2	49.2	256.1

	concentration of iBody 3 (nM)				
	5000	1000	200	40	8
with PSMA	132297	39446	13557	3449	2241
w/o PSMA	156303	46668	12845	2571	2423
Ratio:	0.8	0.8	1.1	1.3	0.9

Supplement references:

[1] P. Šácha, T. Knedlík, J. Schimer, J. Tykvart, J. Parolek, V. Navrátil, P. Dvořáková, F. Sedlák, K. Ulbrich, J. Strohalm, et al., *Angew. Chem. Int. Ed. Engl.* **2016**, *55*, 2356–60.

[2] J. Tykvart, J. Schimer, J. Barinková, P. Pachl, L. Postová Slavetínská, P. Majer, J. Konvalinka, P. Sácha, *Bioorg. Med. Chem.* **2014**, *22*, 4099–4108.
